# Report of the Physical and Mathematical Class of the National Institute of France, on the Question, Proposed to Them by the Minister of the Interior: "Are Those Manufactures Which Emit a Disagreeable Smell Prejudicial to Health?" Read at a Late Sitting of That Body

**Published:** 1806-08

**Authors:** 


*Report of the Physical and Mathematical Class of the National Institute of France, on the Question, proposed to them by the Minister of the Interior : " Are those Manufactures which emit a disagreeable Smell prejudicial to Health?" Read at a late Sitting of that Body. By Messrs.* Guyton Morveau *and* Chaptal.

THE solution of the question, whether the vicinity of certain manufactures be prejudicial to health, must appear the more important, as, by the confidence placed in the opinion of the Institute, it may in future form the ground of judicial decisons, when the suppression of a manufacture and the health of the people are concerned.

This inquiry is become the more necessary, as the fate of the most useful establishments, and even the existence of many arts, have hitherto depended on mere regulations of the police, and as several are driven to a distance from materials, from labour, or from a ready market for their commodities, by the efforts of prejudice, ignorance, or jealousy, continue an unequal struggle against the numberless obstacles opposed to their success. Hence, we have seen manufacturers of acids, of sal ammoniac, Prussian blue, beer, and leather, banished from the precincts of cities, and hence these, as well as similar useful establishments, are still daily complained of by fastidious neighbours, or invidious rivals.

So long as the fate of such manufactures is insecure; so long as an arbitrary legislation can interrupt, suspend, or fetter the exertions of the manufacturer; how can it be expected, that he will have the temerity to embark in projects of this nature? This state of uncertainty, these continual quarrels existing between the manufacturer and his his neighbours, the constant apprehensions he must entertain respecting the fate of his establishment, contract and paralyse his exertions, and slowly impair his property, and spirit of enterprise.

It is then of material importance to the prosperity of the arts, that limits should be fixed, which leave nothing to the will or caprice of the magistrate, which point out to the manufacturer the boundary, within which he may safely exert his industry, and secure to the adjoining proprietors the sure enjoyment of their health, and the produce of their estates. It appears to us indispensably necessary, in order to solve this important question, to take a view of each of those arts, against which the most numerous objections have been urged.

With this view, it may be proper to divide them into two classes. The first will comprehend all those, during the operations of which, gaseous effluvia, the product of putrefaction, or fermentation, that are either unpleasant to the smell, or deemed noxious in their effects, escape into the atmosphere.

The second will include all those where, by the action of fire, various principles are evolved, and diffused in the form of vapour, or gas, of which the inhalation is not only disagreeable, but accounted more or less injurious to health.

In the former, we may rank the steeping of flax and hemp; the manufacture of catgut; slaughter-houses; starch manufactures; tanneries, breweries, &c. In the latter, the distillation of acids, wines, and animal matters; the art of gilding metals; preparations of lead, copper, mercury, &c.

The arts comprehended in the first class deserve particular attention, considered in relation to public health; since the fumes emitted by fermentation, or putrefaction, are, under certain circumstances, confessedly prejudicial to health. The steeping of flax, for example, which is practised in pits, or pools of stagnant water, infects the air, and is fatal to fish. The diseases to which this gives rise are all known and described. It has therefore been wisely ordered, that this operation should be performed without the limits of cities, at a certain distance fiom all habitations, and in waters containing no fish of importance to the inhabitants.

Other operations, which are practised on vegetables, or certain products of vegetation, with the view of obtaining fermented liquors, as in breweries; to prepare colours, as

 in in manufactories of turnsol, archil, and indigo; or to free them from some of their principles, as in starch manufactories, paper-mills, &c. do not appear to us, from their nature, to furnish any cause for uneasiness, on the part of the magistrate. At all events, the fumes, arising from such fermenting substances, can only be dangerous in the immediate vicinity of the vessels and apparatus in which they are contained; they cease to be so, the moment they enter into combination with the open air; a little prudence is therefore only necessary in order to avoid danger. Besides, this cannot affect the inhabitants of neighbouring dwellings; it concerns, and can only injure those employed about the works; so that any law ordering the removal of such works to a distance from all habitations, would, on the part of government, be at once oppressive and unjust, in opposition to the progress of the arts, and not calculated to remedy the evil supposed to be produced.

Some preparations, which are extracted from animal bodies, require their previous putrefaction, as in those connected with the making of cat-gut; but more frequently the animal substances employed in different works are suffered to putrefy by remaining too long in the manufacture, or by reason of too high a temperature. This is particularly the case in the dying of red cotton, where much blood is employed. The effluvium exhaled from this corrupted matter is widely diffused, and forms in the neighbourhood a very disagreeable atmosphere. It would therefore be proper to cause the materials to be frequently renewed, in order to prevent their corruption, and to keep the place clean, so that none of the refuse of the animal matters should be suffered to remain till they become putrid.

In this point of view, slaughter-houses are attended with some inconvenience; but this is by no means of so serious a nature as to demand their banishment without the limits of cities, and their being erected in one place, as projectors are daily suggesting to government. A little attention on the part of the magistrate, to prevent butchers throwing out the blood and offal of the animals, is sufficient to obviate any disgusting and unhealthy effects arising from slaughter-houses.

The manufacture of *poudrette* is now fast gaining ground in all the principal cities of France; and the operation by which night soil is reduced to a state of powder, necessarily diffuses a most disagreeable odour. Works of this kind kind ought therefore to be formed in airy situations, and far from any dwelling; not that we regard the gaseous exhalation from them as injurious to health, but it cannot be denied that it is inconvenient, tainted, disagreeable, and difficult to breathe. On this account, therefore, they ought to be removed to a distance from the habitation of man.

There is one very important observation to be made, respecting the spontaneous decomposition of animal substances, which is, that the exhalations from them appear to be less dangerous, in proportion as the putrefying matters are less humid. In this last case, a considerable quantity of carbonate of ammonia escapes, which imparts its predominant character to the other volatilized matters, and corrects the bad effect of those which would otherwise be deleterious. Thus the decomposition of stercoraceous matter, and of the refuse of the cocons of the silk-worm, in the open air, and in situations the position and inclination of which permit the escape of the liquids, evolve an enormous quantity of carbonate of ammonia, which counteracts the poisonous quality of certain other vapours; whilst these same substances, decomposed in water, or very wet, emit sweetish or nauseous fumes; the breathing of which is attended with extreme danger.

The numerous arts in which the manufacturer produces and diffuses into the air, during the course of his operations, and by the agency of fire, vapours of which the inhalation is more or less disagreeable, constitute the second class which we are to examine. These, which are much more interesting than the former, as well as more intimately connected with the success of national industry, are still more frequently objects of refere ce to the decision of the magistrate; and, on this account, they appear to us deserving of more particular attention.

We shall begin our examination with the preparation of acids.

The acid, against the manufactory of which neighbours may complain, are, the sulphuric, nitrous, muriatic, and acetic. The sulphuric is obtained by the burning of a combination of sulphur and saltpetre. It is very difficult, in this operation, to prevent a smell of sulphureous acid from being diffused round the apparatus in which the combustion is going forward; but in well contrived works this smell is scarcely perceptible in the manufactory itself; the workmen daily breathe these vapours with impunity, and any complaint on the part of the neighbours must be unfounded. When the art of making sulphuric acid was first introduced into France, the popular clamour was very great against these establishments: the smell from the matches, used in our houses, contributed not a little to exaggerate the effect supposed necessarily to follow from the rapid combustion of some hundred pounds weight of sulphur; but this opinion is at present so completely overturned, that we observe many of these manufactories quietly flourishing in the midst of our cities.

The distillation of the muriatic and nitrous acids (spirit of salt and aquafortis) is not more dangerous than the making of sulphuric acid. The whole process is carried on in vessels of earthen ware, or glass, and the chief concern of the manufacturer is undoubtedly to diminish the loss, or volatilization, as much as possible. Nevertheless, whatever attention may be given to the process, the air which is breathed in the manufactory is always impregnated with the peculiar smell of each acid; notwithstanding which, respiration in such situations is free and easy, the workmen are not in the least incommoded by it, and any complaint from the neighbours would be unjust.

Since manufactories of white lead, verdegris, and sugar of lead, have become so numerous in France, vinegar is now more generally used than formerly. In distilling this acid, to render it more fit for some of these uses, a very strong smell of vinegar is diffused, which is not dangerous; but when a solution of lead in this acid is evaporated, the vapours assume a sweetish character, and produce, upon those who are in the habit of breathing them, all the effects peculiar to the vapours of lead itself. Happily, these effects are confined to the men who work in the building, and cannot injure those who reside in the neighbourhood.

Preparations of mercury, lead, copper, antimony, and arsenic, the process of gilding metals, are all somewhat dangerous to those who inhabit and assist in the works; but the effects are confined within the enclosure of the manufactories, and are only dangerous to those concerned in them.

It is an object worthy the attention of chemists to investigate the means of preventing these unpleasant effects. Already much inconvenience has been obviated by the help of chimnies, which convey the vapours out of the reach of respiration. At present, all the attention of administration ought to be confined to directing science towards the improvements of which these processes are susceptible with regard to health.

The making of Prussian blue, the extraction of carbonate of ammonia by the distillation of animal bodies, in the new sal ammoniac works, produce a great quantity of fœtid vapours. In truth, these exhalations are not dangerous to health; but in these times, to be reckoned a good neighbour, it is not enough to be harmless, we must also not be disagreeable. The projectors of these establishments, when about to fix upon a situation, ought certainly to prefer one at a distance from any dwelling; but when the establishment is already formed, we should be careful of advising the magistrate to order its removal. In this case, it will be sufficient to require of the proprietor, that he shall raise his chimnies to such a height as to dissipate, in the air, the disagreeable vapours produced by his operations. This plan is particularly well calculated for the making of Prussian blue; by putting it in execution, one of ourselves has contributed to preserve, in the centre of Paris, one of the most important manufactures of this kind, against which the neighbours had already combined.

In the present Report, we have thought it our duty to confine ourselves to the chief manufactures, against which violent complaints have been preferred at various times and places; and from what has been said, it must be sufficiently apparent, that the vicinity of a few only is prejudicial to health.

Hence, we cannot too seriously advise the magistrates intrusted with the care of the public health and safety, to dismiss those ill-grounded complaints, too often directed against such establishments, which daily threaten the prosperity of the honest manufacturer, retard the progress of industry, and compromise the fate of the arts themselves. The magistrate should be on his guard against the insinuations of a jealous neighbour; he should carefully distinguish between what is only inconvenient or disagreeable, and what is really noxious and dangerous; he should recollect that the use of pit-coal was abolished for a considerable time, under the frivolous pretext of its being unwholesome; he should, in short, be fully sensible, that, by listening to complaints of this nature, not only the establishment of many useful arts in France might be prevented, but that it might be the means of imperceptibly banishing from cities, farriers, carpenters, joiners, braziers, coopers, founders, weavers, and all those whose profession is disagreeable, or inconvenient to his neighbours. For such such trades are unquestionably more disagreeable to a neighbourhood than the manufactures above-mentioned, and the only advantage they enjoy over them is their antiquity. Their right to toleration has been established by time and necessity; and doubtless, when those manufactures, of which we have spoken, become older and better known, they will peaceably enjoy the same advantages in society. In the mean time, we are of opinion, the Institute ought to profit by this circumstance, to place them under the special protection of government, and declare that manufactures of acids, sal ammoniac, Prussian blue, sugar of lead, and of starch, as well as slaughter-houses, breweries, and tan-pits, are not prejudicial to the health of the neighbourhood when properly conducted.

We cannot affirm so much respecting the steeping of hemp, cat-gut manufactures, lay-stalls, and all those works where great quantities of animal or vegetable matter are exposed to humid putrefaction; for, besides the disagreeable smell they exhale, vapours are emitted which are known to prove more or less hurtful.

We should add, that, although the manufactures of which we have been speaking, and which we have considered as not injurious to health by their vicinity, should not be removed, yet the administration ought to exercise over them the most active watchfulness, and consult persons capable of pointing out the most proper means, for preventing the diffusion of the smoke or smell throughout the neighbourhood. This end may be attained by improving the processes, by elevating the outer walls, so as to prevent the diffusion of the vapours, by improving the chimnies, which might be so constructed, that the smoke itself should be condensed, or burnt in the fire-place; by keeping the premises extremely clean, so that no putrefaction shall take place; and by conveying off, into deep pits, all the remains susceptible of fermentation, so as not to become in the smallest degree inconvenient to the neighbourhood.

We must once more observe, that when new establishments are about to be formed for making Prussian blue, sal ammoniac, leather, starch, and all those manufactures in general which emit offensive vapours, or which are in frequent danger from fire or explosion, it would be at once wise, just, and prudent, to declare that these establishments cannot be erected within the limits of cities, and near habitations, without special authority being first obtained for that purpose; and that where the founders of them them do not comply with this indispensible conditon, the removal of such establishments may be ordered without their having a right to demand any indemnity.

The result, then, of our Report is, 1st. That establishments for the making of cat-gut, dung-hills, steeping of hemp, and in general, all those in which a great mass of animal or vegetable matter is heaped up to rot or putrefy, are injurious to health, and ought to be carried on at a distance from cities, and every other habitation. 2dly. That buildings, from which offensive smells are emitted, in consequence of the action of fire, as in the distillation of acids, the making of Prussian blue, and sal ammoniac, prove only dangerous by their vicinity, from want of due precaution; and that the care of administration ought to centre in an enlightened and active superintendence, having for its objects the perfection of the processes employed, the regulation of the fire, and the maintenance of cleanliness. 3dly, That it would become an enlightened and vigilant administration to enact regulations for prohibiting in future, without previous authority, the erection of manufactures in cities, or near to dwelling houses, the vicinity of which is necessarily inconvenient, or dangerous. In this class may be included the manufactures of *poudrette*, tan-pit, starch manufactures, founderies, melting-houses for tallow, slaughter houses, rag-warehouses, manufactures of Prussian blue, varnish, glue, sal ammoniac, potteries, &c.

Such are the conclusions submitted to the Institute by Guyton and Chaptal; and we are subsequently informed, that they were fully approved of by that learned body, and transmitted to the government, with an invitation to adopt them as the basis of its decision.

